# Challenges for controlling bovine tuberculosis in South Africa

**DOI:** 10.4102/ojvr.v87i1.1690

**Published:** 2020-02-27

**Authors:** Luke F. Arnot, Anita Michel

**Affiliations:** 1 Department of Production Animal Studies, Faculty of Veterinary Science, University of Pretoria, Pretoria, South Africa; 2 Bovine Tuberculosis and Brucellosis Research Programme, Department of Veterinary Tropical Diseases, Faculty of Veterinary Science, University of Pretoria, Pretoria, South Africa

**Keywords:** African buffalo, bovine tuberculosis control, cattle, conservation, game farming, *Mycobacterium bovis*

## Abstract

All effects taken together, bovine tuberculosis (bTB) has a long-term detrimental effect on bovine herds and many wildlife species in South Africa. The disease is not only found in domestic cattle but also in African buffaloes and has to date been diagnosed in 21 wildlife species, including several rare and endangered species, thus having a potentially serious effect on conservation and biodiversity. In cattle, bTB is mostly characterised by sporadic outbreaks, but bovine herds chronically infected with the clinical disease are not uncommon. Presently, the recognised bTB control strategy in South Africa is based on ‘test and slaughter’, using the intradermal tuberculin test, followed by the slaughter of animals that have tested positive. Affected herds are placed under veterinary quarantine with movement restrictions until the outbreak is eradicated; this can take several years or last indefinitely if the outbreak cannot be eradicated. The same measures apply to infected buffalo populations, often with no prospect of ever being eradicated. This strategy is neither practical nor viable in the context of a communal farming system and becomes unethical when dealing with valuable wildlife reservoir hosts. Transmission of bTB between wildlife and cattle has been demonstrated and emphasises the need for an effective, affordable and culturally acceptable control strategy to curb the spread of bTB in South Africa. In countries with similar challenges, vaccination has been used and found to be promising for treating wild and domestic reservoir species and may hence be of value as a complementary tool for bTB control in South Africa.

## Introduction

Bovine tuberculosis (bTB) is an economically important and widespread disease in cattle caused by *Mycobacterium bovis* (*M. bovis*) with the potential to establish to certain wild animal species. The organism belongs to the so-called *Mycobacterium tuberculosis* complex, which is characterised by the ability of its members to cause tuberculosis in mammalian species, including humans. *Mycobacterium bovis* has been circulating within European Mediterranean bovine herds from biblical times (Myers & Steele 1969), spreading to Western Europe with the movement of cattle across Europe (Muwonge et al. 2019). Apart from being an impediment to livestock production and human livelihoods, bTB is a threat to the health of wildlife species and people, particularly in developing countries (Ayele et al. 2004; Hlokwe et al. 2016; Michel et al. 2015; Miller et al. 2017a; Renwick, White & Bengis 2007). In South Africa, the zoonotic significance of *M. bovis* is poorly understood, but in light of its soaring HIV statistics bTB can potentially contribute significantly to the human tuberculosis burden (HSRC 2018; Olea-Popelka et al. 2017; Park et al. 2010).

Following the spillover of bTB from cattle to wildlife, African buffalo (*Syncerus caffer*) emerged as the maintenance host, with far-reaching implications for multispecies ecosystems because infected buffaloes act as a source of *M. bovis* to any mammalian species sharing the habitat with them (De Vos et al. 2001). As a consequence, *M. bovis* has infected and been diagnosed in 21 wildlife species (Hlokwe et al. 2019; Michel et al. 2015), including near-threatened, rare and endangered wildlife species such as white and black rhinoceros and wild dog (Higgitt et al. 2019; Miller et al. 2016, 2017b, 2018). In addition to buffalo, greater kudu (*Tragelaphus strepsiceros*) has shown the potential to act as a maintenance host and most probably responsible for introducing *M. bovis* into a conservation area (Michel et al. 2009). Warthogs (*Phacochoerus africanus*), a social species that show predominantly lung and intestinal tuberculous lesions, are considered potential bTB reservoir hosts in ecosystems supporting high population densities (Michel et al. 2015); however, conclusive evidence is difficult to obtain in large reserves where other reservoir species are present – an assessment which is also true for lions (*Panthera leo*) (Michel & Van Helden 2019).

A future negative impact on wildlife conservation efforts is considered inevitable unless the spread of bTB can be addressed urgently and effectively. The aim of this article was to review the current challenges and opportunities in controlling bTB in the domestic and wild reservoir species in South Africa.

## Bovine tuberculosis in cattle and current control measures

Bovine tuberculosis spread to South Africa and other colonies with the importation of cattle from Europe in the early 1800s and was first diagnosed in a bovine in South Africa in 1880 (Henning 1956; Myers & Steele 1969). Importation of large numbers of cattle mostly from Europe and also Australia and South America before the existence of bTB screening or control measures led to the increase in the number of cattle diagnosed with tuberculous lesions at major abattoirs in South Africa (Cousins et al. 2004). For this reason, bTB received attention as a major livestock disease as far back as 1911 under the *Diseases of Stock Act* and later when the bTB Control and Eradication Scheme was launched in 1969. During the 1970s, there was a clear emphasis on establishing bTB-free commercial cattle herds, and a herd accreditation scheme was strongly promoted (Michel, Sibanda & De Klerk-Lorist 2019). The control interventions were highly successful and led to a drastic decrease in bTB prevalence in the commercial cattle farming sector where the herd prevalence decreased from 11.8% in 1971 to 0.39% in 1995 (Michel et al. 2008). The decentralisation of State Veterinary Services and the re-prioritisation of disease control efforts coupled with budgetary constraints severely hampered bTB control in subsequent years, with the number of cattle tested annually plummeting in all provinces from the late 1990s onwards (Cloete 2015). As a consequence of these and other challenges in controlling this disease, bTB has slowly been re-emerging in commercial cattle production throughout South Africa (Michel et al. 2008, 2019). In the communal cattle farming sector, the bTB prevalence had remained unknown until recently where studies have revealed a high variability in animal prevalences ranging from very low (< 0.5%) to high (>15%) (Musoke et al. 2015; Sichewo, Etter & Michel 2019).

Surveillance of cattle herds for bTB is the responsibility of the provincial veterinary services, whereby budget allocations and availability of human resources determine surveillance activities. Cattle owners who want their animals tested can do so at their own expense. However, once an outbreak of bTB is suspected or confirmed through meat inspection at abattoirs or *ante mortem* testing, the control measures laid down in the *Bovine Tuberculosis Manual* of the Bovine Tuberculosis Scheme, issued by the Department of Agriculture, Forestry and Fisheries, are to be applied (DAFF 2016). State veterinarians assume complete control of all diagnoses and control measures. This means infected farms are placed under quarantine, and cattle are tested using the comparative or single intradermal tuberculin test, also referred to as tuberculin skin test (TST). Test-positive cattle are branded and slaughtered, and the remaining herd is subjected to a repetition of these procedures at 3-month intervals until the herd is cleared of any suspect or positive reactor animals during which time the quarantine can be lifted. Compensation may be paid for condemnations only.

## *Mycobacterium bovis* infection in wildlife and current control approaches

In South Africa, *M. bovis* has affected wildlife species for many decades and was first identified in a greater kudu in the Eastern Cape in 1928, followed by identification in a duiker (*Sylvicapra grimmia*) in 1929 (Paine & Martinaglia 1928). These cases were presumed to be isolated incidents. The first confirmed case in a buffalo within a recognised wildlife reserve in South Africa occurred when a buffalo was found to be infected within the Hluhluwe–iMfolozi Park (HiP) in 1986, followed by a lion in 1992 and kudus, bushpigs (*Potamochoerus larvatus*) and baboons (*Papio ursinus*) in the same reserve in 1998 (Buss 2015). The HiP reserve was only fenced in the 1950s, and it is presumed that bTB was introduced by spillover from adjacent communal cattle to buffalo during co-mingling prior to HiP being fully fenced (Hlokwe et al. 2011; Wadge 2007).

The first suspected case of *M. bovis* infection within the Kruger National Park (KNP) was an impala that died in 1977, but the diagnosis was never confirmed by culture (Buss 2015). This unconfirmed case was thought to be an isolated incident, until the first index case was confirmed in a young buffalo bull in the southern section of the park in July 1990 (Bengis et al. 1996). A bTB-positive dairy herd on a farm located south of the Crocodile River was reported in the 1950s, and other outbreaks on cattle farms in the same area were reported in 1982, 1983 and 1984 (Cloete 2015). The Crocodile River forms the southern boundary of the KNP and was not fenced at the time. Buffaloes were known to cross the river at night to graze on the opposite bank (Bengis et al. 1996), thus coming into contact with cattle on infected farms adjoining the KNP.

Soon after this index case in 1990, it was found that only buffalo herds in the southern-most part of the KNP were infected with bTB. There was a drive to contain the spread of bTB from infecting herds located further north within the reserve (Caron, Cross & du Toit 2003). Several proposals were put forward to prevent bTB from spreading, including creating a buffalo-free zone using a fenced off cordon and intensive culling of buffalo within the cordon to protect unaffected herds, implementing a form of metaphylaxis vaccination, or a combination of these options, as opposed to letting the bTB take its natural course (Caron et al. 2003; De Vos et al. 2001). One such proposal was to create a buffalo-free zone by culling all buffaloes starting at the Olifants River in the south, then extending for a 20-km zone in a northerly direction. Any buffalo within the 20-km zone would be culled, and then the culling zone would be extended southwards to eliminate all buffaloes to the south of the river, thus eliminating the spread to herds north of the buffalo-free zone (Bartlett 1997). However, during the planning phase, it was found that bTB had already spread to buffalo herds to the north of this zone (Bartlett 1997), and the plan was abandoned. By 2006, buffalo herds in the far northern areas of the KNP were found to be infected (Buss 2015). In 2008, buffaloes within the Gonarezhou National Park in Zimbabwe tested positive, and the same strain circulating within the Kruger buffalo was isolated, implying that bTB from the KNP had spread across South Africa’s northern border into Zimbabwe (De Garine-Wichatitsky et al. 2010). It was ascertained by molecular characterisation of bTB strains that the KNP had been infected by a single *M. bovis* parent strain (SB0121), suggesting a single introduction into the KNP (Hlokwe, Van Helden & Michel 2013, 2014; Michel et al. 2009), which was most likely from the infected dairy farm adjacent to the southern border of the KNP during the 1950s.

The Madikwe Game Reserve (MGR), located in the far northern regions of the North West Province, was home to a very valuable disease-free^1^ buffalo herd comprising outstanding genetic stock. This herd originated from 53 buffaloes imported back into South Africa from European and American zoos (De Klerk-Lorist 2015). In 2012, prior to an auction, three of the 51 buffaloes tested positive for the disease and were confirmed to be infected with *M. bovis* (Hlokwe et al. 2016). Initial epidemiological investigations failed to reveal how bTB had been introduced into MGR, but genetic characterisation by spoligotyping and Variable Number of Tandem Repeat (VNTR) typing made it possible to trace back the *M. bovis* isolate to wildlife in Kwazulu-Natal (KZN). It is strongly suspected that amongst the estimated 8000 antelopes translocated into MGR, the *M. bovis* strain was introduced by another bovid species from KZN. As can be seen from the case above, even under strict biosecurity with regard to cattle and buffalo movements, introduction of *M. bovis* into an unaffected area by other bovid species is a reality.

In 2014, a bTB prevalence survey amongst communal cattle within the Mnisi area, located adjacent to the western border of the KNP, revealed that 0.39% of these communal cattle were infected with the KNP strain of *M. bovis* (Musoke et al. 2015). This finding proved for the first time that spillback from wildlife reservoir hosts in KNP back into adjacent communal cattle was indeed happening. This finding has far-reaching consequences for the national bovine herd in that wildlife reservoir hosts will continue to spread *M. bovis* amongst themselves in an uncontrolled manner and will constitute a persistent risk to cattle herds that are in close proximity. In a most recent study in northern KZN close to but not adjoining the HiP, communal cattle were found to be co-infected with different *M. bovis* strains all of which were shared with buffaloes in HiP (Sichewo et al. 2019).

With the increasing human population pressure at the wildlife or livestock interface of conservation areas, such as the KNP and HiP, spillover and spillback incidents will continue to pose a significant risk to cattle unless bTB can be controlled within the wildlife reservoir host. Vice versa, in the absence of bTB control in cattle, exposure of wildlife hosts to *M. bovis* continues to exist making spillover inevitable.

The application of control measures for *M. bovis* infection in buffalo presents a dilemma for State Veterinary Services because the *Bovine Tuberculosis Manual* has been written exclusively with a view on controlling bTB in cattle and thereby ignoring the need to manage infected buffalo herds (and potentially other wildlife species) on commercial ranches (Michel et al. 2015, 2019). While the presence of *M. bovis* prohibits the open trade in wildlife species in general, pre-movement testing for bTB is only mandatory for buffaloes. In essence, movement of buffaloes from disease-free populations is subject to a negative comparative TST. Should the TST yield inconclusive results, the test must be repeated after 3 months. Particulars for test interpretation and management of infected herds are similar to those for cattle herds and are set out in the veterinary procedural notice (VPN) (DAFF 2017). For all practical purposes, infected buffalo ranches are placed under quarantine indefinitely, unless the owner can provide satisfactory evidence that *M. bovis* has been successfully eradicated from the population.

## Challenges in the control of bovine tuberculosis in cattle

The introduction of *M. bovis* strains into South Africa with infected cattle from Europe during colonial times promoted a high genetic diversity of strains, representing the European (EU)-1 and possibly the EU-2 clonal complexes (Michel et al. 2008). EU-1 is primarily circulating within cattle in the Republic of Ireland and the United Kingdom, and former colonies of Britain but is absent from the rest of Africa (Smith et al. 2011). EU-2 strains are dominant in the Iberian peninsula from where they were possibly disseminated by international trade (Rodriguez-Campos et al. 2012). In South Africa, two major interventions could contribute to the decline of the presence of bTB in cattle, namely, the rinderpest epidemic at the end of the 19th century as well as the implementation of the Tuberculosis Scheme in 1969 specifically aimed at eradication of bTB. In spite of the drastic reductions in the number of infected cattle, the diversity of the prevailing bTB strains remained high, with spoligotype SB0130 being most prevalent as shown in a study in 2014 (Hlokwe et al. 2014). Over and above, compared to a previous study using *M. bovis* isolates dating to the end of the 20th century (Michel et al. 2008), an increased interspecies spread of bTB has been recorded indicating the transmission of *M. bovis* between cattle and wildlife (Hlokwe et al. 2014; Sichewo et al. 2019). The persistence of highly diverse *M. bovis* strains in the national cattle herd is not only testimony to an impaired efficacy of the prevailing control strategy but may also point towards a potential selection for highly successful *M. bovis* outbreak strains that are able to evade conventional control measures.

The ‘test and slaughter’ approach forms the foundation of bTB eradication in developed countries. Looking at the reasons behind the success of this control strategy, we find five essential requirements that must be in place: a uniform (commercial) cattle farming system, stringent test interpretation (accepting an ‘overkill’ of cattle), financial compensation for cattle slaughtered, adequate government resources guaranteeing completion of the eradication campaign and the absence of an *M. bovis* wildlife reservoir (Amanfu 2006; Smith et al. 2011). The significance of the latter is demonstrated by the inability of countries including, but not limited to, the United Kingdom, Ireland, Spain and New Zealand to eradicate bTB from the cattle population in spite of large financial commitments (Reviriego Gordejo & Vermeersch 2006).

In South Africa, buffaloes are highly effective maintenance hosts of bTB, which facilitate the persistence and continued horizontal spread not only to other wildlife species but also to cattle beyond the border of game and veterinary control fences, thus jeopardising bTB control in cattle (Musoke et al. 2015; Sichewo et al. 2019). It is practically impossible to eradicate bTB where there is a possibility of contact between buffalo and cattle herds (Phepa, Chirove & Govinder 2016).

In 2015, the number of cattle in South Africa was estimated to be 13.9 million. Of these, 58% reside in the commercial sector while about 42% of the national herd are kept in communal farming systems (DAFF 2015, 2017). Cattle belonging to different small-scale communal farmers mix together over common grazing lands. These cattle congregate periodically in large numbers at communal dip tanks for dipping and other basic veterinary procedures, and at communal watering points. The aggregation of large numbers of communal cattle at these points provides an ideal opportunity for the spread of bTB amongst these cattle.

Communal cattle, in contrast to commercially farmed cattle, constitute more than a commercial value to their owners; the number of cattle owned by a communal cattle farmer is a measure of wealth and social status within the community. Apart from being a source of animal protein, cattle also serve as a source of cash for emergency expenditure, draught power, fertiliser, hides and are highly important for cultural practices (Meltzer 1995; Scholtz et al. 2008). The control of bTB or any other controlled disease that requires the compulsory removal of animals amongst communal cattle herds therefore imposes unique and difficult to accept challenges on the owners and their families which are currently not dealt with in a culturally sensitive manner.

From a technical point of view, the TST has severe shortcomings in the communal farming sector:

- Impractical: testing of cattle is conducted at the crush pen of the relevant dip tank facility and disrupts the regular dipping activity on the day of tuberculin injection. In addition, cattle owners have to return their cattle to the dip tank after 72 h for the reading of the skin reactions. As a consequence, high rates of farmer non-compliance and a resultant poor testing coverage are observed.
- Unethical: in contrast to a developed country setting where a certain ‘overkill’ or wastage of healthy animals because of the limited specificity of the TST is acceptable or affordable, this approach is to be rejected on ethical grounds in a developing country where food security is not guaranteed.
- Uncertain quality: for the ‘test and slaughter’ strategy to be a successful control method, the TST must be performed by competent personnel with functioning, calibrated testing equipment. In reality, financial constraints and lack of trained personnel can render the TST a non-reliable method generating test results of questionable quality without checks and balances.
- Unsustainable: quarantine measures instituted in a communal farming area mean that all farmers, whether their herds are infected or not, are collectively denied market access for their animals, rendering the control strategy unsustainable and unacceptable to farmers. For all practical purposes, the quarantine remains in place indefinitely because eradication of bTB is difficult or impossible to achieve in the absence of earmarked financial resources and adequate compensation for slaughtered animals.

The control of bTB in many developing countries, including South Africa, is severely hindered because of general government fatigue of chronic, neglected, difficult-to-control livestock diseases affecting primarily marginalised farming communities. As a result, very few communally owned cattle are routinely tested for bTB. The frequently poorly regulated movement of animals and lack of systematic and verifiable livestock identification also complicate the issue (Amanfu 2006).

## Challenges in the control of *Mycobacterium bovis* infection in African buffalo

South Africa is blessed with an amazing array of wildlife species, which is one of the greatest attractions for tourism in South Africa, contributing billions of rands into the South African economy each year, with tourism contributing 2.9% of the total Gross Domestic Product (GDP) of South Africa in 2016 (Statistics SA 2018). The wildlife industry, together with ecotourism, hunting and game sales grew by 20.3% per year in the past 15 years and created over 140 000 permanent jobs (Oberem 2016). However, there is a possibility that *M. bovis* infection can pose a severe threat to the wildlife diversity, on the one hand, and the income from tourism, on the other hand.

African buffaloes play an important ecological role as bulk grazers and key prey species. Their commercial use is based on their iconic status for ecotourism, game farming and hunting safaris. The wide utilisation explains their popularity, resulting in a widespread distribution throughout conservation areas and privately owned game operations (Michel & Bengis 2012; Prins 1996). Whether they are free-ranging or are actively translocated, through their cardinal role as a maintenance host of bTB, buffaloes also serve as a disease amplifier and effective disseminator of *M. bovis*, and thus ongoing surveillance and efficacious control measures are needed for buffaloes and possibly other species sharing the same habitat.

A case of *Mycobacterium orygis* infection in a buffalo was diagnosed in 2012 (Gey van Pittius et al. 2012) and again in a different herd in 2017 (Verrynne, unpublished data), indicating the presence of this Mycobacterium Tuberculosis Complex (MTBC) organism in South Africa and its ability to cause positive TST reactions. In spite of extensive follow-up testing in the second case over a period of 2 years, no indication for the spread of the organism within the herd was found.

For the time being, the bTB control measures, including the TST prescribed for cattle, have been adopted for buffaloes, which bear several inherent impediments to ensuring the quality of the diagnosis made and the health and welfare of the buffalo during testing:

- Immobilisation causes severe stress and potential fatalities
- Direct and severe consequences of the ‘test and removal’ control approach:
  ■ Unethical culling of buffalo as a result of the limited test specificity
  ■ Undue financial losses to buffalo owners
- Boma confinement may predispose buffaloes to shedding and transmission of *M. bovis*
- Quality of test results may be compromised by:
  ■ The current lack of sufficient validation data required to set adequate interpretation criteria
  ■ The absence of trained state veterinary officials competent to conduct and interpret the TST

Performing the TST in buffaloes is a difficult and expensive process, as the animals need to be confined in a boma where they are chemically immobilised twice within 72 h to perform the TST. The interferon-gamma assay (Bovigam^®^), an alternative bTB test that can be used as an ancillary test at the discretion of the state veterinarian, mitigates the need to confine the buffalo in bomas for 3 days but still necessitates immobilising buffaloes in order to obtain blood samples (Michel et al. 2011). In addition, the Bovigam^®^ assay requires the whole blood sample to reach a laboratory for stimulation within 8 h, which is not always possible in remote locations.

## The way forward?

The future success of controlling bTB in cattle and wildlife populations in South Africa will depend on an effective, affordable, practical, sustainable, and culturally and ethically acceptable control programme in cattle and buffalo that is potentially expandable to other wildlife species. To achieve this goal, it will be important for the South African government as the custodian of livestock animal health, conservation agencies as well as producers’ organisations to enter into consultations and private–public partnerships to address strategies for the control of bTB in a stakeholder-sensitive manner. The diverse spectrum of economic, cultural and species settings in which bTB control must be effectively applied renders a ‘one-size-fits-all’ control strategy ineffective and unrealistic. In specific commercial settings, ‘test and slaughter’ with compensation through industry-driven initiatives can be a viable control strategy, while vigilant surveillance at population and individual animal level (the latter at slaughter and pre-movement), community-based animal health care, increased education and involvement of communal cattle farmers with regard to suitable and locally relevant bTB control could be implemented in communal small-scale herds. In addition to all of the above, a vaccination strategy for domestic and wildlife reservoir species could afford the opportunity to significantly reduce the prevalence of diseased animals and limit the onward spread of the disease (Buddle et al. 2018).

## Can vaccination contribute to bovine tuberculosis control in South Africa?

In developed countries with an effective bTB control programme in cattle and a bTB wildlife reservoir host, it has been shown that eradication in livestock depends on concurrent and successful control in the wildlife reservoir (De Lisle et al. 2002). In South Africa, the situation is more complex as bTB in cattle is poorly controlled or uncontrolled in some areas and is a potential source of infection to valuable healthy wildlife populations in conservation areas and also in commercial game operations. Therefore, vaccination of both cattle and wildlife reservoir species should form part of a comprehensive strategy to combat bTB in South Africa.

The aim of vaccinating domestic or wild *M. bovis* reservoir species is to induce an immune response that ideally protects the animals against infection. However, incomplete protection, meaning infection not being prevented in the vaccinate, is still highly beneficial if the infection cannot progress to a disease, resulting in significantly reduced disease severity (Palmer & Thacker 2018). Several TB vaccine approaches have been explored in humans and in animals; compared to subunit and DNA vaccine candidates, Bacillus Calmette-Guerin (BCG) appears to be the most successful candidate in animals. The BCG vaccine is presently the only vaccine registered for the control of tuberculosis in humans and the most common vaccine used experimentally in the control of bTB in cattle. After a series of controlled vaccination experiments, a recent vaccination trial in free-ranging cattle in New Zealand showed that low-dose BCG protected cattle from either becoming infected or from developing gross lesions with a trial efficacy of 86% (Nugent et al. 2018). If applied systematically, vaccination is expected to reduce *M. bovis* prevalence and can contribute significantly to an integrated bTB control in cattle using lethal and non-lethal approaches (Buddle et al. 2011, 2013). However, one drawback of BCG vaccination is that it causes a positive TST reaction for several months, which appears to drop to a vaccine reactor rate of 10% after about 9 months post vaccination (Chambers et al. 2014). The development of DIVA (differentiating infected from vaccinated animals) tests has been explored and may mitigate this problem in future (Buddle et al. 2013).

In wild reservoir hosts, vaccination holds the potential to slow down interspecies transmission to vulnerable wildlife species and also to cattle, which is a major benefit where other control measures are not feasible or not desirable. Bacillus Calmette-Guerin has also been researched in affected wildlife, including European badgers (Chambers et al. 2014), white-tailed deer (Palmer, Thacker & Waters 2007), European wild boar (Gortazar et al. 2014) and brush tailed possums (Buddle, Wedlock & Denis 2006; Tompkins et al. 2009) with beneficial but variable effects. De Klerk et al. (2010) found that BCG was able to reduce the number of lesions in experimentally infected yearling African buffalo although not statistically significant. The use of the live attenuated BCG vaccine also seems attractive because it requires only a single dose, which makes its application in wildlife more practical.

On the other hand, the use of a live vaccine in animals destined to enter the human food chain could raise concerns of potentially negative implications for immunocompromised patients (Norouzi et al. 2012) as BCG organisms may persist in the edible tissues (Palmer et al. 2012). The use of inactivated vaccines against bTB would therefore be preferred because of being environmentally safer and more stable under field conditions, thereby causing no disease in non-target wildlife species where safety challenges have not been conducted when compared to the live BCG vaccine (Garrido et al. 2011). Recent studies using an inactivated *M. bovis* vaccine have demonstrated protective responses comparable or superior to BCG in a number of wild and domestic animal species, indicating promising prospects for the future use of these vaccines. These inactivated *M. bovis* vaccines provided good protection in European wild boar (Garrido et al. 2011), red deer (Lopez et al. 2016), cattle (Jones et al. 2016; Van der Heijden et al. 2017) and sheep (Balseiro et al. 2017). When used for vaccination of cattle, the same inactivated *M. bovis* vaccine induced strong and sustained cell-mediated and humoral immune responses, significantly higher than the control group in response to vaccination. These cattle were challenged with live BCG vaccine, and although not statistically significant, recovery of BCG after challenge was lowest in the group vaccinated with inactivated *M. bovis* as compared to BCG and unvaccinated control animals (Van der Heijden et al. 2017).

In general, more stringent requirements for vaccine use have been postulated for cattle than for wild life. It should be emphasised that this strongly depends on the context in which the vaccine is to be applied. For a vaccine to be considered successful in cattle in epidemiological settings in which conventional control measures are either absent or not implemented because they are deemed impractical, unethical, unaffordable, wasteful or culturally unacceptable, the hallmark of full protection against infection is not justified, similar to the requirements of a wildlife vaccine (Buddle et al. 2006). Under those circumstances, vaccination can be regarded as the best tool available to perform the task (Buddle et al. 2018). The same principle of local context also applies to the significance of interference of a vaccine with future diagnostic testing. While it is not a concern for wildlife without commercial use, in countries where a high number of wild animals are traded, determining the bTB status by TB testing is relevant. For this reason, it is of utmost importance to define the expectations and evaluate the limitations of a vaccine in the local context of farming systems and environments.

In summary, bTB control in South Africa faces unique challenges within the livestock sector, the wildlife industry and in conservation areas alike, which cannot be successfully addressed using conventional resource-intensive control measures based on culling alone. Vaccination offers a valuable adjunct control tool that has been demonstrated to effectively protect animals from TB disease and can slow down its transmission in multiple species. Prior work conducted in South Africa has established infection models for both cattle and African buffalo, which form a suitable foundation for future vaccine efficacy trials under controlled and field conditions (Buddle et al. 2013; De Klerk et al. 2006, 2010; Van der Heijden et al. 2017).

## Conclusion

It is essential that bTB be well-controlled within South Africa because of the risk it poses to livestock production, commercial game operations, wildlife conservation and human health and livelihoods. However, current approaches to bTB control are not or less effective than required to fulfil the mandate of the Tuberculosis Scheme. The underlying reasons for this inefficacy are manifold and economic, political, technical, social and ethical in nature. To improve the overall bTB control, a new strategy is urgently needed, which takes into account affordability, feasibility and cultural acceptability of the measures to be implemented. Vaccination offers a promising strategy as an integral part of the bTB control in South Africa.

## Footnotes

1 Free from foot-and-mouth disease, corridor disease, brucellosis and bovine tuberculosis.
